# Staff perception on including students with physical disabilities at a South African university

**DOI:** 10.4102/ajod.v13i0.1347

**Published:** 2024-03-15

**Authors:** Mashudu R. Mphohoni, Martha Geiger, Surona Visagie, Mashudu Manafe

**Affiliations:** 1 Department of Occupational Therapy, School of Health Care Sciences, Sefako Makhatho Health Sciences University, Pretoria, South Africa; 2 Department of Global Health, Faculty of Medicine and Health Sciences, Stellenbosch University, Cape Town, South Africa; 3 Department of Human Nutrition and Dietetics, School of Healthcare Sciences, Sefako Makgatho Health Sciences University, Pretoria, South Africa

**Keywords:** students with physical disabilities, inclusive higher education practises, health care sciences, inclusion policy, disability unit, SWPD enrollment

## Abstract

**Background:**

International and local policy frameworks on disability promote inclusive higher education practices for students with disabilities (SWD). However, the actual application of these frameworks concerning students with physical disabilities (SWPD) in any School of Health Care Sciences (SHCS) is uncertain in South African universities.

**Objectives:**

This study aimed to explore the perceptions of academic and admission staff on the inclusion of SWPD in SHCS at a South African university. The study was carried out at a University of Health Sciences in South Africa.

**Method:**

A qualitative study in which respondents (*n* = 12) were interviewed in depth about their perceptions on the inclusion of SWPD in the SHCS. Thematic analysis was used in the data assessment.

**Results:**

The results revealed three main themes: policy discourse, environmental effects on inclusion and SWPD enrolment. Respondents reported the lack of a disability inclusion policy and disability unit to support SWD in general. The respondents also noted that there were environmental challenges that could potentially affect the inclusion of SWPD in SHCS study programmes. Respondents also indicated that there was no SWPD enrolment as the university’s current inclusion and/or quota system does not include SWD.

**Conclusion:**

The findings of the study showed a lack of disability inclusion policy, environmental challenges and lack of SWPD enrolment. Based on the study findings, it can be concluded that inclusion of SWPD at this university may be negatively influenced.

**Contribution:**

The study findings contribute to the field of disability and the inclusion of SWPD in higher education institutions (HEIs).

## Introduction

The implementation of inclusive higher education remains poor in higher education institutions (HEIs), and the actual enrolment figures for students with disabilities (SWD) are generally low (under 1% of students enrolled) (Department of Higher Education [DHET], 2018). In an overview of student demographics at eight medical schools in South Africa, the authors of Van der Merwe et al. (2016) reported on race and gender, but not disability, despite having selection policies and practices for undergraduate health sciences study programmes that aim to redress and achieve student diversity. The Wits Disability Rights Unit (DRU) report shows that a total percentage of SWD in the health sciences faculty was 2.2% (Wits DRU 2022). However, there is a dearth of literature in relation to enrolment statistics in health sciences faculties. It is assumed that excluding disability could be the reason for the under-representation of SWD in medical schools and health sciences faculties, and this could negatively influence the recruitment and selection of students with physical disabilities (SWPD) in the School of Health Care Sciences (SHCS).

An enabling environment with accessible buildings like the lecture halls or theatres, libraries and toilets as well as modes of transport is important to promote the smooth integration of SWPD into health sciences study programmes (Chiwandire & Vincent, 2017; Moodley & Mchunu, 2019). Although some accessibility improvements were realised globally, Ntombela (2013) noted that many South African universities are still disabling as they consist of grassy and uneven pathways and inaccessible buildings, where SWPD rely on others for access. Physical access restrictions remain the most crucial challenge experienced by SWPD in HEIs (Engelbrecht & De Beer, 2014; Moriña, 2017). Chiwandire and Vincent (2017) added that poorly designed buildings physically exclude SWPD from participating in higher education. Therefore, the physical environment should allow for the accommodation of SWPD.

According to South Africa’s *Education White Paper 6: Special Needs Education* (Department of Education [DoE], 2001), curricula in most institutions create the most significant barrier to learning and inclusion for SWPD. The authors, Bunbury (2018) and Dalton et al. (2019), suggested that HEIs should adopt an inclusive curriculum designed according to inclusive models that minimise the hindrance to learning and participation by SWPD. As highlighted by Rankin et al. (2010), those professional study programmes in which curricula consist of placement components to ensure that students are able to demonstrate practice competency regardless of their impairment or disability present the greatest barriers, e.g. clinical placement requirements in health sciences study programmes. Authors such as Rankin et al. (2010), Bialocerkowski et al. (2013) and Moodley and Mchunu, (2019) criticised the clinical component requirements as they are designed as a ‘one size fits all’ model or the ‘fitness to practice’ concept for able-bodied students without possible accommodations for SWPD.

Contrary to findings by Mayat and Amosun (2011) that showed that academic staff were willing to admit and accommodate SWPD, some faculty members in HEIs still display negative attitudes towards SWPD (Engelbrecht & De Beer, 2014; Moriña, 2017). Ntombela (2013) added that SWPD still experiences exclusion based on other peoples’ perceptions as they are viewed as people who need help. Ntombela (2013) was of the opinion that negative attitudes towards SWD in general have continued to have an impact on inclusive education initiatives by those who want transformational changes. It is critical for HEIs to create awareness among staff members on the diversity of the student population to achieve or realise transformational changes (Joubert & Martins, 2013). Tillman et al. (2015) postulated that it is necessary to have positive attitudes and increase the exposure of historically under-represented people like those with disabilities to health sciences study programmes if their access to those careers is to be realised. This means that the recruitment drive in communities should target schools and organisations for people with disabilities (PWD) to make them aware of and develop an interest in health sciences careers. Tillman et al. (2015) asserted that expanding the representation of SWPD in health sciences study careers is possible, thus increasing diverse enrolment.

One of the gaps identified in the literature includes the reported lower rates of admission of students with physical disabilities in most HEIs. Health care sciences consist of different rehabilitation groups with different functions. Including SWPD in health care sciences groups will ensure a diverse workforce that will enable patient-centredness in the treatment of people with disabilities (Meeks & Moreland, 2021). In addition, innovative ideas, including the use of technology, can be explored and implemented to make individuals with disabilities part of the health care system. There is a dearth of literature on the inclusion of SWPD, and the findings of this study will add value to the existing literature, influencing positive perception on the inclusion of SWPD. Therefore, the purpose of this study was to explore the perceptions of academic and admission personnel about the inclusion of SWPD in the SHCS of a South African university.

## Research methods and design

### Study design

The study used a qualitative descriptive study design to describe the perceptions of academic and admission personnel involved in the selection and admission of students regarding the inclusion of SWPD in the SHCS study programmes.

### Setting

The study was carried out at a Health Sciences University in South Africa that consists of the Schools of Medicine, Pharmacy, Health Care Sciences, Oral Health Sciences, as well as the School of Science and Technology.

### Study population and sampling strategy

The study used purposeful, nonprobability sampling in which respondents from the SHCS and Central Admission and Enrolment Departments were included by virtue of their expertise in student selection matters (Crossman, 2020). The following departments were included: Occupational Therapy (OT), Physiotherapy (PT), Speech Language Pathology and Audiology (SLPA), Human Nutrition and Dietetics (HND), Nursing and all administrative staff members from the Central Admission and Enrolment Department were selected to be part of the sample. The SHCS and the Central Admission and Enrolment Departments were selected because they comprise departments involved in the rehabilitation of PWD and the selection of students, respectively. The researcher approached the departments individually and provided information about the research. Each head of department was requested to relay the information to their staff members about the research, and the names of those who were willing to participate in the study were communicated to the researcher. Thereafter, respondents were approached by the researcher to form part of the study sample. The recruitment process took 1 month. The number of individuals who volunteered to participate dictated when recruitment into the study ceased. When recruitment ceased, there were 12 respondents, which formed the study sample size.

### Data collection

A semi-structured interview schedule was used to collect data. The interviews were recorded. Data were collected over 2 months, from November 2018 to December 2018. The interviews were conducted in English and lasted about 30 min. The interviews were conducted privately at a place convenient for each participant within the research setting. The researcher made sure that all aspects of the research study were thoroughly described. Direct quotes from the interviews were used to present the findings.

### Trustworthiness

To ensure trustworthiness, the author read the transcripts multiple times to ensure understanding of the data was maintained. A digital recorder was used to ensure the rigour. Peer debriefing sessions were held between the author and coauthors during the analysis to agree on the study’s data and findings.

### Data analysis

The audio-recorded interviews were transcribed verbatim. A list of codes and definitions was kept, allowing researchers to track how codes are used to make sense of the data. To become familiar with the data, the researcher read the transcripts carefully and repeatedly. Thematic analysis was carried out by identifying, analysing and reporting patterns (themes) within the data. The responses were further analysed using inductive techniques by which relevant responses relating to the inclusion of SWPD were coded from the data. Initial codes were generated by coding data related to practices, and similar codes were grouped into themes and subthemes for interpretation. The results were then discussed in coded themes and subthemes of the topic under discussion. Finally, the themes and findings were presented through a narrative with data quotes.

### Ethical considerations

The study was carried out according to the Declaration of Helsinki and was approved by the Stellenbosch University Health Research Ethics Committee. The approval study number is Ref: # S18/05/114. The researcher provided the respondents with the information leaflet and the informed consent form. All respondents gave their informed consent before participating in the study.

## Results

Twelve respondents comprised the sample of the study. The sociodemographic data were used to describe the sample characteristics and did not necessarily influence their perceptions. Most of the respondents were females (*n* = 10) and most of the respondents (*n* = 8) had working experience of 1–10 years. Respondents were between 30 years and 65 years (Table 1).

**TABLE 1:** Sociodemographic characteristics of the respondents (*N* = 12).

Sociodemographic characteristics	*n*	%
**Age (years)**		
30–45	5	42
46–65	7	58
**Sex**		
Male	2	17
Female	10	83
**Working experience (in years)**		
1–10	8	66
11–20	2	17
21 and above	2	17

## Themes

Three themes emerged from the data: policy discourse, environmental effects on inclusion and enrolment of SWPD. Details of the themes are shown in Table 2.

**TABLE 2:** Themes and sub-themes.

Themes	Sub-themes
Policy discourse	Disability inclusion policy Disability Office or Unit
Environmental factors	Physical access Clinical curriculum requirements Attitudes
Enrolment of SWPD	Recruitment Selection Admission

SWPD, students with physical disabilities.

## Policy discourse

### Disability inclusion policy

The findings indicated that the respondents were unaware of any policy that addresses the inclusion of SWD in general at this university.

‘…To my knowledge, we do not have a policy on inclusive higher education …, so I have not seen a policy on disability yet.’ (Participant 4)

‘I am not aware of the current policies regarding inclusive higher education in this university.’ (Participant 2)

One respondent also highlighted the impact that not having a disability policy has on the handling admission issues relating to SWD in general:

‘A year or two ago there was a deaf girl whose mom was phoning me and emailing me … nobody was prepared to talk to them. I know she wanted to do medicine … but nobody could help me and I really tried. Therefore, there was no written document [*policy*] to give guidance. I really tried to make appointments with the Dean of that school and nothing happened; they could not even have an interview with that girl and her mom, and they really wanted to talk. And that was very difficult for me because I feel everyone needs a chance … even just the chance to them talk to and explain to them.’ (Participant 8)

### Disability unit or office

Furthermore, the findings indicated that there was no disability structure, such as a disability unit or office, to support SWD in general:

‘This university has no Disability Desk or Unit and has not embraced disability in terms of students and staff.’ (Participant 1)

### Environmental factors

#### Physical access

The respondents indicated that the physical campus environment (lecture halls, libraries, laboratories, kitchens, walking paths and transport) is not conducive to inclusion in terms of access and mobility. Although the clinical placement areas or sites (hospitals, clinics, old age homes and special schools) should be accessible, respondents highlighted that they are not accommodating or user-friendly to people with mobility problems:

‘The challenge we have now is the accessibility of the therapy rooms, test rooms, lecture rooms and things like that because the university has not designed the university to accommodate SWPD.’ (Participant 1)

‘The lecture halls and the library can be a challenge … Clinical placement areas … is a challenge. Transportation to a placement area … is also a problem if the transport cannot accommodate them.’ (Participant 5)

#### Clinical curriculum requirements

The respondents were of the opinion that SWPD might not be unable to meet the clinical demands or requirements of the curriculum to provide safe and ethical services to patients. These requirements are part of the minimum training standards set out by the Health Professions Council of South Africa (HPCSA):

‘As a student, you have to be trained in all aspects of rehabilitation … and that is where the difficulty lies. … if a physically disabled person cannot perform, say, for instance, neuro rehabilitation, issuing them with a certificate that says: “I have graduated as a therapist”; it is unfair and unethical to do so ….’ (Participant 3)

‘Wheelchair users can have serious physical problems when they have to do evaluations and measure a patient who is upright …’ (Participant 5)

‘In terms of curriculum, the barrier would be the clinical requirements because we expect them to perform physical conditions, where they have to perform transfers and treat patients on standing frames. We had a wheelchair-bound student whose hands could not straighten out … he would not be able to meet the requirements from second year … we tried to refer him to a programme … but he refused … It took us a year to get that student off to another programme on another Campus.’ (Participant 12)

#### Attitudes

The respondents noted that people’s attitudes could affect inclusion:

‘I think … we still look at PWD as people who want our service, not as people who can be our peers …’ (Participant 1)

‘So there are many barriers …’ having the stigma of being different from the rest of the students.’ (Participant 7)

‘… We normally see disabled people differently and we don’t treat them like normal people … the level of awareness … from peers, is not enough.’ (Participant 9)

‘I think attitudinal barriers can play a big role in making sure they are not maybe included, … we all have our prejudices and I think we pre-judge and think these ones are not going to make it before we even give them a chance.’ (Participant 10)

However, some respondents reported that it could be possible to include SWPD with reasonable accommodations to facilitate their inclusion at this university despite the above barriers:

‘… we should include them. But then we should keep in mind how we can accommodate these students in our departments and in the clinical facilities where we train them off campus.’ (Participant 4)

‘… I believe that students with physical disabilities should be admitted. … we can find means and ways of training them differently … without compromising the requirements for the main degree.’ (Participant 10)

Respondents with a positive attitude towards inclusion hinted that the response towards inclusion at this university could be reactive:

‘… Our university is reactive and they are not proactive. If a student becomes physically disabled, then that they can be reactive and implement things to help the student …, but we are not proactive, so we wait for that day … then we can modify the environment to include PWD.’ (Participant 1)

‘… the only time we will change is when we actually get a SWPD. So, currently we don’t see the need to modify our buildings …, but when we have someone disabled coming, it will be the trigger to say that now we need some adjustments.’ (Participant 4)

### Enrolment of students with physical disabilities

#### Recruitment

According to the respondents, potential applicants with disabilities are not recruited to apply for admission to this university. They pointed out that the marketing of health sciences programmes was not extended to PWD:

‘… we usually go out and market the professions in schools and the communities. I don’t think we are more open to going to schools with physical disabilities to make them aware of our degrees.’ (Participant 1)

‘I do not think we have got enough awareness in the disability community about our programmes as a whole …’ (Participant 12)

#### Selection

Respondents noted that the final selection criteria or the quota system only addressed race, gender, students with prior degrees and foreign students, but that this criterion did not address disability:

‘… I know for sure that we do not look at people with disabilities. We look at race … gender. The application form has your quota … so many percentages of women and men … different races, but it does not say anything about people with disabilities.’ (Participant 1)

‘… our inclusion criteria or practises we are looking at … quota; … regarding race, gender, and foreign nationals … we do not look at people with disabilities.’ (Participant 4)

#### Admission

Some of the respondents pointed out that they were not aware of any SWPD enrolled at the study site:

‘I don’t think we have any inclusive practises, I have been here since 2002 … I have never seen a student who had physical disability admitted in our programme.’ (Participant 1)

‘In the 5 years that I have been here; I have not seen a student with a physical disability admitted into the programme.’ (Participant 4)

However, other respondents had experiences with SWPD in the past, who later succeeded in their studies and graduated:

‘I had the experience of a CP diplegic student some years back. He graduated … now he is a very successful therapist.’ (Participant 3)

‘We had one student here this year who had severe scoliosis … A couple of things had to be adapted for him … He was included in all other activities and now he is a graduate.’ (Participant 7)

The respondents were aware of a possibility that SWD could be admitted to the study site without disclosing their disability status, perhaps for fear of being discriminated against:

‘My experience is that students do not disclose, even when the university application form requires you to disclose. I was on a Senate Committee for people with disabilities … I was given a mandate to do an audit of how many students live with disabilities. I went to the student administrator and they said: “nobody would disclose” because they are afraid they would be discriminated and not allowed to continue. I said how do I know and they said “no student would write that on their file” …’ (Participant 12)

## Discussion

The study aimed to qualitatively explore the perceptions of academic and admission staff on the inclusion of SWPD in SHCS at a South African university. Most of the respondents were women. However, age and gender did not influence on the perceptions related to the inclusion of SWPD in a HEI. The work experience of the respondents contributed to their perception about the subject under investigation. The respondents in this study believed that the university had no disability inclusion policy and no disability office or unit to support SWD. This is so despite the availability of the international and local policy framework on disability inclusion in HEIs such as UNCRPD (UN, 2007), WPRPD (DSD, 2016) and the Strategic Policy Framework on the PSET System (DHET, 2018). The idea that the university has no disability inclusion policy and disability office or unit to support SWD in general is contrary to the intentions of the South African DHET to formulate, implement and monitor disability-related policies and guidelines in PSET institutions (DHET, 2018). However, it should be noted that there is a paucity of studies on the inclusion of students with disabilities in general in medical and health care sciences study programmes, especially in developing countries like South Africa.

The findings of the study are contrary to what the authors of Ramaahlo et al. (2018) reported that each HEI in South Africa is required to have its disability unit to promote and support inclusive higher education. Furthermore, Ntombela (2013) is of the view that a Disability Office in an institution is meant to support SWPD who experience barriers to learning and development. Without a disability policy to rectify past imbalances, inclusion efforts may not be achieved. Furthermore, without a disability structure, institutions lack procedures or directives to include or support SWPD. The implication, therefore, is that those who work in the admission department may find it difficult to handle queries related to the inclusion of SWPD.

The respondents identified environmental issues that could potentially affect the inclusion of SWPD in the SHCS study programmes. These issues were related to physical access, clinical curriculum requirements and attitudes. Due to physical access constraints or poorly designed physical environments, SWPD are still excluded from participating in higher education (Chiwandire & Vincent, 2017; Engelbrecht & De Beer, 2014). The authors Chiwandire and Vincent (2017) stressed that accessibility of buildings as well as improvement in modes of transportation should be prioritised to facilitate participation of SWPD in learning and related activities. As highlighted by Rankin et al. (2010), participation in health care programmes by SWPD was found to pose considerable barriers due to its professional clinical placement components, where students are required to demonstrate clinical practice competency. Moodley and Mchunu (2019) and Bialocerkowski et al. (2013) agreed and highlighted that the curricula of health sciences programmes do not promote equal access due to the design of clinical components as a ‘one size fits all’ model concept.

The findings show that people’s attitudes pose a significant challenge that could affect inclusion. The findings are in line with the findings of Ntombela (2013), who reported that SWPD still experiences exclusion based on the perception of others due to their physical conditions, as people who need help. However, respondents who showed a positive attitude believe that it is possible to include SWPD with reasonable accommodations to facilitate their inclusion at this university. In a case study report on a SWPD in a professional physiotherapy education programme with functional limitations in standing, sitting, lifting and bending, Francis et al. (2007) noted that if special equipment is provided, students can participate in classroom and clinical education activities. Similarly, Jackson et al. (2011) recommended the provision of special equipment, extra time for tasks or special positioning as part of accommodating SWPD in health sciences.

Some of the study respondents indicated that they had no experience or awareness of SWPD, while other respondents did experience SWPD in the past. There is the possibility that there were no SWPD enrolled in the SHCS at the study site at the time of the study. This is in line with the enrolment figures of SWD in South African universities, which were less than 1% of the total enrolled students enrolled (DHET, 2018). Furthermore, having no enrolment in SWPD is contrary to the recommendations of international and local policy initiatives to increase enrolment in SWPD enrolment in higher education (DSD, 2016; UN, 2007).

The study findings revealed that the respondents are of the opinion that potential SWPD might not be aware of the study programmes that the university offers, as they are not recruited to apply. Moodley and Mchunu (2019) confirmed this finding when they cited a lack of SWPD recruitment in most Nursing Education Institutions (NEI) than lack of internal policy guidelines. Authors such as Bialocerkowski et al. (2013) view the lack of recruitment of SWPD as an element of discrimination, especially when there could be evidence that they were not actively recruited or given information. Therefore, access to this career in health sciences by SWPD is not being promoted. However, Tillman et al. (2015) asserted that expanding the representation of SWPD in health care sciences careers is possible if there is an increase in diverse enrolment. Essentially, the marketing of the study programmes should be extended to the disabled community to realise their inclusion in HEIs.

The respondents noted that the quota system for student selection does not include those with disabilities. However, the study findings revealed that other additional inclusion criteria are based on gender and other variables, except disability, as noted by the respondents. Just like recruitment practices, not including the general population of SWD in the selection criteria means that inclusive higher education can also be largely seen as discrimination against SWPD (Bialocerkowski et al., 2013).

The study findings suggest that there may be SWD admitted to the study site who did not disclose their disability status. These findings are consistent with those reported by Moodley and Mchunu (2019) in which some SWD did not disclose their disabilities for fear of being excluded from the nursing programme. Similarly, those with a negative experience of being excluded from a study programme after revealing their disability might be reluctant to disclose their disability fearing that it would place them at a disadvantage (Moriña, 2017; Lourens & Swartz, 2016). In contrast, Ndlovu (2019) and Moodley and Mchunu (2019) at their study sites found no evidence against discrimination of disabled applicants in terms of application procedures, even though the applicants had indicated their disability. A disability inclusion policy is paramount in addressing such issues and should, therefore, explicitly state how prospective SWPD will be recruited and selected to ensure representation within the student body population.

### Strengths and limitations

The strengths of the study are that new information is presented in the field of disability. While most studies focused on the inclusion of SWD in general at HEIs without specifying the study programmes, this study specified the type of disability in health sciences study programmes. The authors are of the opinion that the findings herein can generate more curiosity into how inclusion of SWPD in health sciences study programmes can be done. The limitation of the study is that since a qualitative method of enquiry was used, the findings cannot be generalised as perceptions differ from individual to individual. The study was carried out only in one of the five schools at the university. Future studies should include people from other schools who are involved in the selection process.

## Conclusion

The study explored and provided information on the perceptions of academic and admission personnel about the inclusion of SWPD in the SHCS at a South African university. The inaccessibility of a disability inclusion policy at this university could affect the enrolment of SWPD. Although the inclusion of SWPD does occur in some instances, policy, proactive planning around physical spaces and curriculum requirements, as well as harnessing the positive attitudes of staff, can improve inclusion in the study setting. The environment should form the foundation of disability inclusion and should be able to promote the smooth integration of SWPD into SHCS programmes. Once the disability inclusion policy, the enabling environment and enrolment of SWPD are in place, a disability office can be established to support SWPD at the university. One of the benefits of including students with disabilities is that it may foster empathy and concordance among individuals living with disabilities in terms of addressing health care disparities. Thus, the findings of the study will be of great benefit to the DHET to mainstream disability in HEIs. The findings of the study are critical to the Department of Social Development as custodians of ensuring the rights of people living with disabilities including higher education as a right.
